# Surface Properties and Morphology of Boron Carbide Nanopowders Obtained by Lyophilization of Saccharide Precursors

**DOI:** 10.3390/ma14123419

**Published:** 2021-06-20

**Authors:** Dawid Kozień, Piotr Jeleń, Joanna Stępień, Zbigniew Olejniczak, Maciej Sitarz, Zbigniew Pędzich

**Affiliations:** 1 Faculty of Materials Science and Ceramics, AGH University of Science and Technology, 30 Mickiewicz Av., 30-059 Kraków, Poland; pjelen@agh.edu.pl (P.J.); msitarz@agh.edu.pl (M.S.); 2 Academic Centre for Materials and Nanotechnology, AGH University of Science and Technology, 30 Mickiewicz Av., 30-059 Kraków, Poland; jstepien@agh.edu.pl; 3 Institute of Nuclear Physics, 152 Radzikowskiego St., 31-342 Kraków, Poland; zbigniew.olejniczak@ifj.edu.pl

**Keywords:** boron carbide, saccharides, lyophilization, freeze drying, NMR, XAS

## Abstract

The powders of boron carbide are usually synthesized by the carbothermal reduction of boron oxide. As an alternative to high-temperature reactions, the development of the carbothermal reduction of organic precursors to produce B_4_C is receiving considerable interest. The aim of this work was to compare two methods of preparing different saccharide precursors mixed with boric acid with a molar ratio of boron to carbon of 1:9 for the synthesis of B_4_C. In the first method, aqueous solutions of saccharides and boric acid were dried overnight at 90 °C and pyrolyzed at 850 °C for 1 h under argon flow. In the second method, aqueous solutions of different saccharides and boric acid were freeze-dried and prepared in the same way as in the first method. Precursors from both methods were heat-treated at temperatures of 1300 to 1700 °C. The amount of boron carbide in the powders depends on the saccharides, the temperature of synthesis, and the method of precursor preparation.

## 1. Introduction

Boron carbide (B_4_C), due to its specific properties (high hardness, low density, high melting point, high elastic modulus, etc.) [1,2], has been widely used in many applications such as in polishing as an abrasive, in ball mills as a neutron absorber and as a neutron shield, and in boron neutron capture therapy (BNCT). In BNCT research, nuclear reactors or accelerators generate thermal neutrons, which are captured by a variety of nuclei, but the probability of capture by an isotope of boron (^10^B) is much higher than that of the capture of another isotope. The first studies of synthesized boron carbide (B_4_C) started when Henri Moissan obtained boron carbide from the reduction of diboron trioxide (B_2_O_3_) by magnesium (Mg) in the presence of carbon (C) [1]. Since 1899, boron carbide has been synthesized using various methods. The synthesis methods determine the properties of the products, morphology, and purity of obtained B_4_C [3,4]. The methods of boron carbide synthesis can be classified as carbothermic reduction [3,4,5,6,7], magnesiothermic reduction [8], synthesis from elements [9], vapor phase reaction [10], synthesis from polymer precursors [11], liquid phase reaction [12], ion beam synthesis [13], and vapor-liquid-solid (VLS) growth [14]. Carbothermal reduction is commonly used in industry to obtain B_4_C as a source of boron (boric acid (H_3_BO_3_) or boron oxide (B_2_O_3_), less commonly) and as a source of carbon fine crystalline graphite or petroleum coke. The reaction is carried out at a temperature between 1700 and 2000 °C under a protective atmosphere (Ar) [15]. Powders obtained from carbothermal reduction are strongly agglomerated and aggregated, so they require intensive crushing and grinding so that they become suitable for further use. The main problem is to reduce the cost of synthesis; many researchers have attempted to lower the synthesis temperature using various organic carbon precursors such as phenolic resin, citric acid, polyvinyl alcohol (PVA), and carbohydrates (cellulose, glucose, and sucrose) with boric acid (H_3_BO_3_) [16]. When the boron carbide is synthesized from an organic precursor, the main problem during synthesis is the removal of water from precursors, which avoids the aggregation of particles in precursors, thereby significantly affecting the size and morphology of the boron carbide after synthesis.

Freeze-drying, also known as lyophilization or cryodesiccation, is a method of removing water by the sublimation of ice crystals from frozen material. Lyophilization is an effective method of drying materials without harming them and is commonly used mainly in the food and pharmaceutical industries [16,17,18]. Freeze-drying is also an effective method of removing water from the precursor obtained by the mixed solvent of boric acid and saccharides.

Previous work by our research group [16] only focused on synthesized boron carbide from different types of saccharide precursors as the carbon precursor in carbothermal reduction and studied the influence of the used saccharide precursor with a molar ratio of carbon to boron of 9:1 on the morphology of the obtained boron carbide powders (B_4_C). In the present work, we aimed to verify the idea that the freeze-drying of mixed precursors (boric acid and different saccharides) has an important influence on the morphology and obtained size of boron carbide after synthesis. During the freeze-drying of saccharide precursors mixed with boric acid, water can be removed from the precursor without heat treatment. The second idea presented in the article focuses on how the type of precursor used and the molar ratio of boron to carbon in precursor influence the obtained morphology and aggregation of the powders.

## 2. Materials and Methods

Powders of boron carbide were obtained from boric acid (H_3_BO_3_), and saccharides, including glucose, fructose, dextrin, and hydroxyethyl starch (HES), were all obtained from Sigma Aldrich (St. Louis, MO, USA) (99% pure). In the first method, boric acid (H_3_BO_3_) and mono- or polysaccharides were dissolved in distilled water in a molar ratio providing a boron to carbon ratio in the final powder of 1:9. After mixing the precursor in distilled water, the solutions were prepared as described in our earlier article [16], and then, they were dried overnight at 90 °C in a vacuum oven to obtain them in solid states. In the second method, the powders of boron carbide were prepared using a similar procedure as in the first method, but the only difference was that the solutions of boric acid mixed with different saccharides were freeze-dried to remove water from the precursor’s powders. All obtained precursors from both methods were pyrolyzed at 850 °C for 1 h under argon flow. The pyrolyzed powders were placed in graphite crucibles and heat-treated at a temperature ranging from 1300 to 1700 °C for 1 h under the argon flow. Changes in saccharide precursors were identified by infrared spectroscopy analysis.

The FTIR measurements of both precursors and boron carbide materials were carried out using a Bruker Vertex 70 v spectrometer (Billerica, MA, USA). The standard KBr pellet method was employed, and 128 scans in the range of 4000 to 400 cm^−1^ were accumulated with a resolution of 4 cm^−1^. Raman spectroscopy measurements were recorded to determine the presence of carbon and carbon hybridization in the precursor. A WITec Alpha 300 M+ spectrometer (Wissenschaftliche Instrumente und Technologie GmbH, Ulm, Germany) equipped with a 488 nm diode laser along with a 600 grating and a Zeiss 100× objective lens (Carl Zeiss AG, Oberkochen, Germany) was used. Such combination provides a laser spot of 661 nm in diameter. The power of the excitation source was set to prevent sample degradation. Each sample was measured in five spots with an accumulation of 2 scans, 120 s each, in each spot. Obtained sets of spectra were then averaged. To analyze the local structure in the near-surface region of the obtained precursors, both pyrolyzed and synthesized at 1700 °C, X-ray absorption spectroscopy (XAS) in total electron yield (TEY) detection mode was used. The measurements were performed using the PEEM/XAS beamline of the Solaris National Synchrotron Radiation Centre, Cracow, Poland [19]. PEEM/XAS beamline is a bending magnet-based beamline, providing the soft X-ray energy range (150 to 2000 eV) and equipped with plane grating monochromator of resolving power, E/ΔE > 4000. Samples in the form of powder on carbon tape were measured at boron K edge at room temperature. The carbon tape contribution was subtracted from the collected spectra, and they were normalized to a unit step by subtracting the constant value fitted in the preedge region and then dividing by the constant value fitted in the postedge region. The energy scale was calibrated so that the positions of characteristic spectral features of reference B_4_C were at the same energy values as in [20]. The beginning of absorption starts at 189 eV. On the spectra obtained from the boron edge, we can distinguish four bands from the π* excitation state: (A) 190.9 eV, (B) 191.7 eV, (C) 192.3 eV, and (D) 193.7 eV and three bands from the σ* excitation states: (E) 196 eV, (F) 200 eV, and (G) 204 eV. The obtained spectra were compared with the spectra obtained for boron carbide, amorphous boron, hexagonal boron nitride (h-BN), and cubic boron nitride (c-BN) and boron carbide heated under vacuum conditions to 1000, 1400, 1700, and 1900 K [20,21,22].

The phase composition and the size of the crystallites of the final powders were determined by an X-ray analysis. HighScore Plus software (Version 4, Malvern Panalytical, Malvern, UK) was used to analyze the data. The Scherrer formula was used to calculate the crystallite size, and Rietveld analysis was used to qualitatively analyze the obtained powders. The phase composition of the final powders was determined by an X-ray analysis (XRD) with an Empyrean diffractometer (PANalytical) (Empyeran, Panalitycal, UK) using Cu-Kα1 radiation. The difference between boron carbide obtained from recrystallized and freeze-drying saccharide precursors was observed with a FEI Nano SEM200 (FEG) scanning electron microscope (FEI Company and SU-70, Hitachi, Hillsboro, OR, USA). The measurements were conducted in high vacuum conditions at an accelerated voltage of 10–15 kV with a backscatter electron detector (BSE). The samples were coated with a carbon layer. High-resolution, solid-state ^11^B MAS-NMR spectra were measured using the APOLLO console (Tecmag) and a 7 T, 89 mm superconducting magnet (Magnex) (Tecmag Inc., Houston, TX, USA). A Bruker HP-WB high-speed MAS probe (Billerica, Massachusetts, USA) equipped with a 4 mm zirconia rotor and a KEL-F cap was used to spin the sample at 10 kHz. The resonance frequency was 96.11 MHz, and a single 2 µs RF pulse, corresponding to a π/4 flipping angle in the liquid, was applied. The acquisition delay in accumulation was 1 s, and 256 scans were acquired. The parts per million frequency scale referenced to the ^11^B resonance of 1 mol H_3_BO_3_.

## 3. Results and Discussion

**Figure 1:**
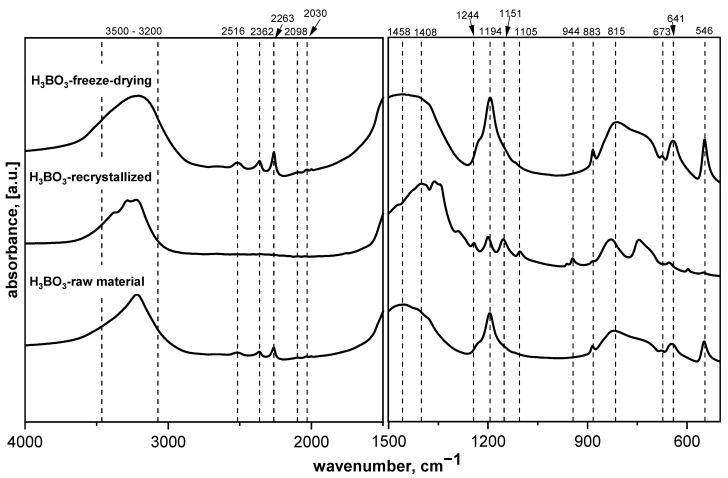
MIR spectra of the raw material dried at 90 °C and freeze-dried boric acid (H_3_BO_3_).

FT-IR measurements in the middle-infrared range (MIR) were recorded to analyze the substrates (saccharides and boric acid) to determine the influence of the precursor preparation process. Figure 1 shows the MIR spectra of the as-obtained starting material (H_3_BO_3_).

Figure 1 presents the MIR spectra of raw boric acid as well as recrystallized and freeze-dried materials. The raw and freeze-dried H_3_BO_3_ are similar, with changes occurring mainly in the O–H stretching and bending range (approximately 3200 and 1640 cm^−1^, respectively). Changes in these spectral regions suggest that molecular water was absorbed after the freeze-drying process. The spectrum of boric acid after the recrystallization process indicates that structural changes occurred. Upon reducing the half-width of the bands, as well as the appearance of new ones, some changes can be observed. One of such changes is the appearance of an additional band responsible for vibrations of O–H groups in the B–O–H system at approximately 3284 cm^−1^, as well as a more distinct band characteristic of the vibration of molecular water at approximately 3388 cm^−1^. In addition, we can clearly see two bands at approximately 1158 and 1198 cm^−1^, which are characteristic for B–O–H bending vibrations, which arose from splitting the band at approximately 1190 cm^−1^. The appearance of the bands at around 1248 and 1104 cm^−1^, the splitting of the 1500–1300 cm^−1^ range into smaller component bands, and the bands appearing below 500 cm^−1^ clearly indicate that the material was oxidized after recrystallization and B_2_O_3_ was formed. A similar situation occurred with the other materials—glucose, fructose, sucrose, and HES. The process of recrystallization, as well as freeze-drying, leads to structural changes, such as amorphization or the appearance of adsorbed water [17].

H_3_BO_3_ → HBO_2_ + H_2_O ↑ (1)

HBO_2_ → ½ B_2_O_3_ + ½ H_2_O ↑ (2)

H_3_BO_3_ → ½ B_2_O_3_ + 3/2 H_2_O ↑ (3)

**Figure 2:**
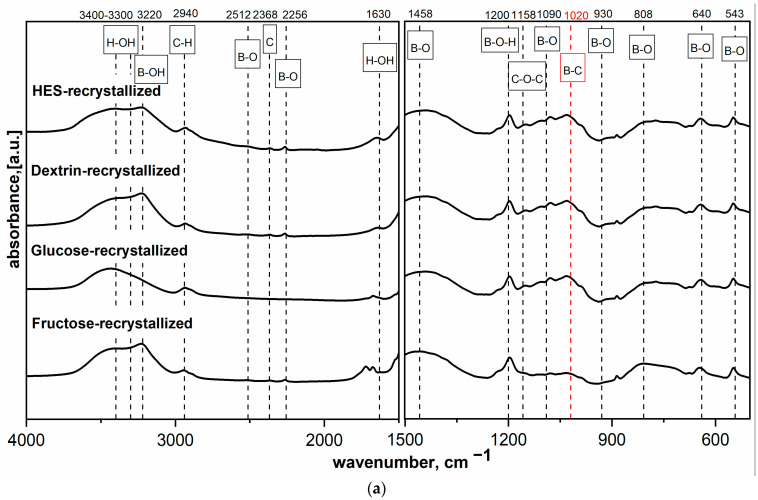

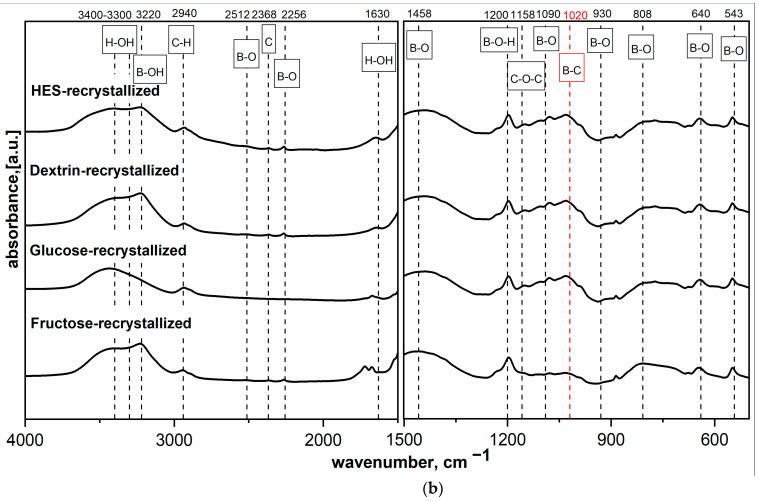
MIR spectra of the different saccharide precursors mixed with boric acid with a molar ratio of boron to carbon of 1:9: (**a**) dried at 90 °C and (**b**) freeze-dried.

When the temperature increases, the dehydration of boric acid occurs, which is related to the weight change and dehydration of boric acid from water (Equations (1)–(3)). The same situation was observed when precursors of saccharides were mixed with boric acid and lyophilized. The effect of lyophilization and recrystallization on the precursors used was significant, showing differences in the spectra obtained from the same precursor but using different methods of preparation. Figure 2a,b shows the MIR spectra of HES precursor mixed with H_3_BO_3_ at different molar ratios and dried at 90 °C or freeze-dried. The findings agree with previous results [16] and indicate that components react with each other, which manifested in the appearance of a new band at 1020 cm^−1^. The band at 1020 cm^−1^ is described in the literature as the band corresponding to B-C bond vibrations. This hypothesis was confirmed by previous research on spectral analysis based on the analysis of the position of band corresponding to the B-C vibrations present in covalent organic frameworks [22] and amorphous boron carbide [16,19]. The increase in boron content in the precursor sample caused a significant increase in the intensity of bands characteristic for B-O and B-OH bond vibrations. Figure 2a,b shows that typical bands for B-O bonds occurred at 1458 and 1090 cm^−1^; this was the expected and desired effect, which was to create the B-C bond already at the precursor preparation stage and thus positively affect the final product.

**Figure 3:**
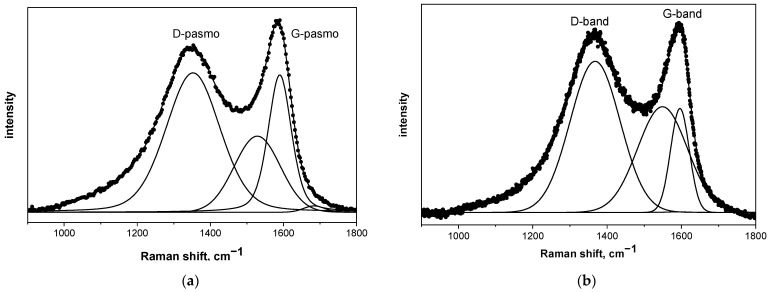
Deconvolution of the Raman spectra of (**a**) HES and (**b**) precursor prepared from a mixture of lyophilized HES and boric acid both pyrolyzed at 850 °C.

The structure of the pyrolyzed precursor at 850 °C was determined using three methods: Raman spectroscopy, nuclear magnetic resonance spectroscopy (NMR), and X-ray absorption spectroscopy (XAS). In all cases, only two characteristic bands were visible on the spectra, which were attributed to the structure of carbon: the G-band at approximately 1600 cm^−1^ and the D-band at around 1350 cm^−1^. The G-band indicates tensile vibrations in the plane of the sp^2^ carbon structure and the D-band is a characteristic of the symmetrical vibration of a breathable hexagonal carbon ring [23]. To compare the differences between the samples according to the Ferrari and Robertson diagram [21,22,23], the absolute intensity of the D-band to the G-band (ID/IG) ratio was calculated. To determine the carbon phase structure, the obtained Raman spectra were subjected to a deconvolution process using Bruker OPUS software and the Levenberg–Marquardt algorithm. The results of this process are presented in Figure 3 and Table 1. These measurements revealed that in the case of pure saccharides, regardless of their type, the I_D_/I_G_ ratio was lower than one, and the position of the G-band fell on approximately 1600 cm^−1^. According to the Ferrari scheme, every analyzed result indicates that carbon occurs in sp^2^ hybridization and forms local regions with a graphite-like structure [16,24,25,26,27]. In each case, the presence of boric acid in the pyrolyzed mixture caused a significant increase in the I_D_/I_G_ value. This effect indicates that the graphite-like structure formed during the heat treatment of the precursors is more defective than with pure saccharide, which is in the agreement with our previous paper [16]. This is due to the boron carbide being formed and, more specifically, the B-C/B-O-C bonds [16]. The I_D_/I_G_ ratio of both recrystallized and freeze-dried samples is similar, which was expected and indicates that both methods produce almost identical carbon-containing materials from a spectroscopic point of view.

**Figure 4:**
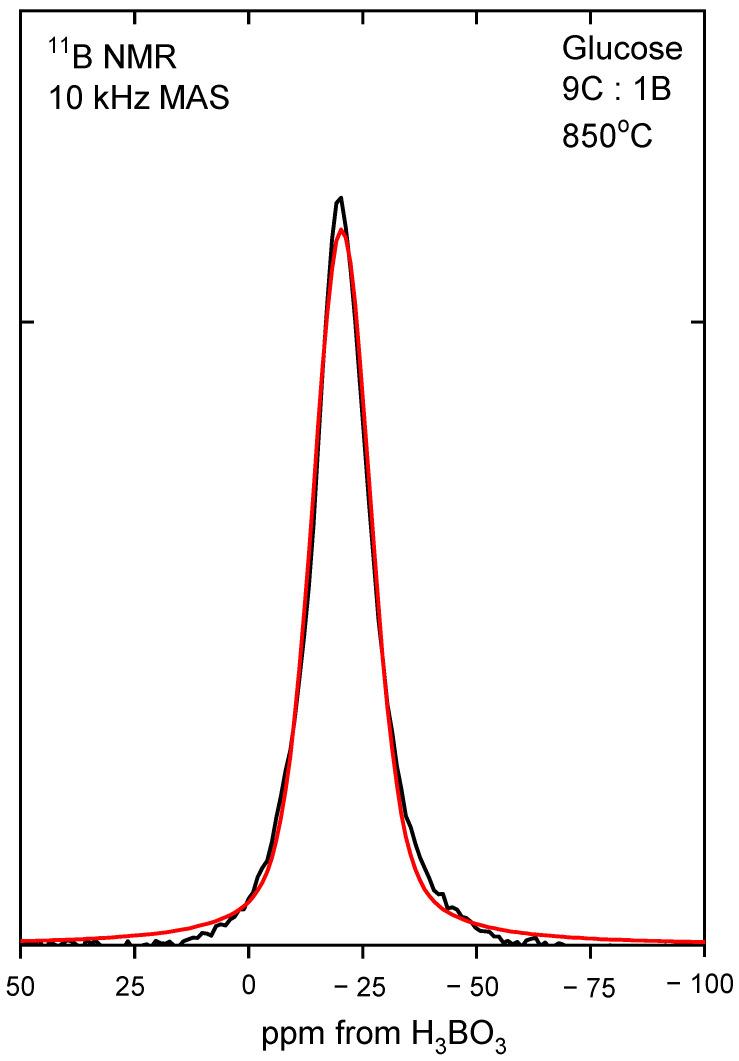
An example of a deconvoluted ^11^B MAS NMR spectrum of fructose mixed with boric acid in a molar ratio of 9 C:1 B and then recrystallized.

In order to determine the boron coordination, ^11^B MAS NMR measurements were performed. All the obtained spectra were deconvoluted, and an example of recrystallized glucose is presented in Figure 4, whereas obtained data are presented in Table 2. The results show that in all of the samples, boron had a tetragonal coordination, indicating that the B4C structure was obtained. A typical FWHH (full width at half height) of the NMR line was about 16 ppm, and the line position varied by 4 ppm. These small variations were within the experimental uncertainties and the accuracy of numerical fitting procedure. It can be, therefore, concluded that the chemical environment and symmetry of the site occupied by the boron atom was essentially not affected by the precursor type and the preparation method.

**Figure 5:**
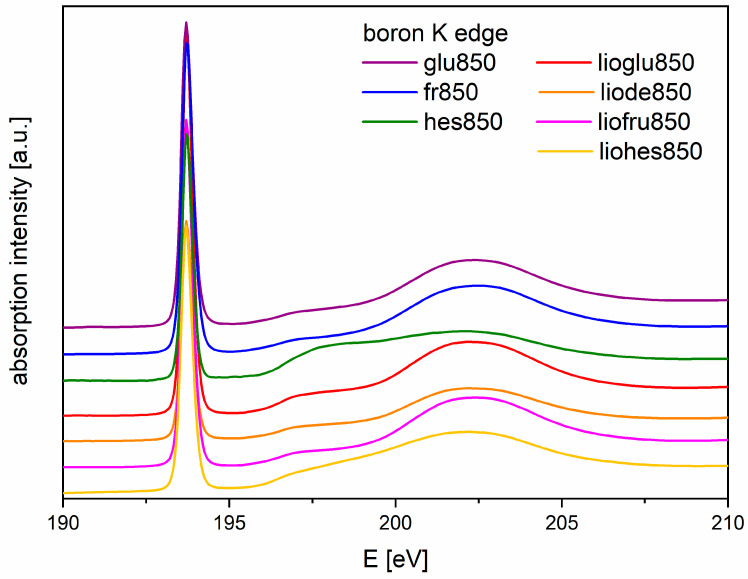
Boron edge spectra obtained from the PEEM/XAS research line for recrystallized and freeze-dried precursor after pyrolyzing at 850 °C.

Figure 5 shows that boron edge spectra for both methods after pyrolyzing (recrystallized or freeze-dried) obtained from the research line PEEM/XAS have the same shape, except for two spectra obtained for the powders where the precursor saccharide was HES. Despite these differences in all the precursors, there was a D-band located around 193.7 eV, which indicated the presence of boron oxide in the precursors. The use of saccharides for the carbothermic reduction of boron carbide probably increased the melting point of boric acid and influenced the formation of new bonds between the saccharide and boric acid, which affected the synthesis of boron carbide.

**Figure 6:**
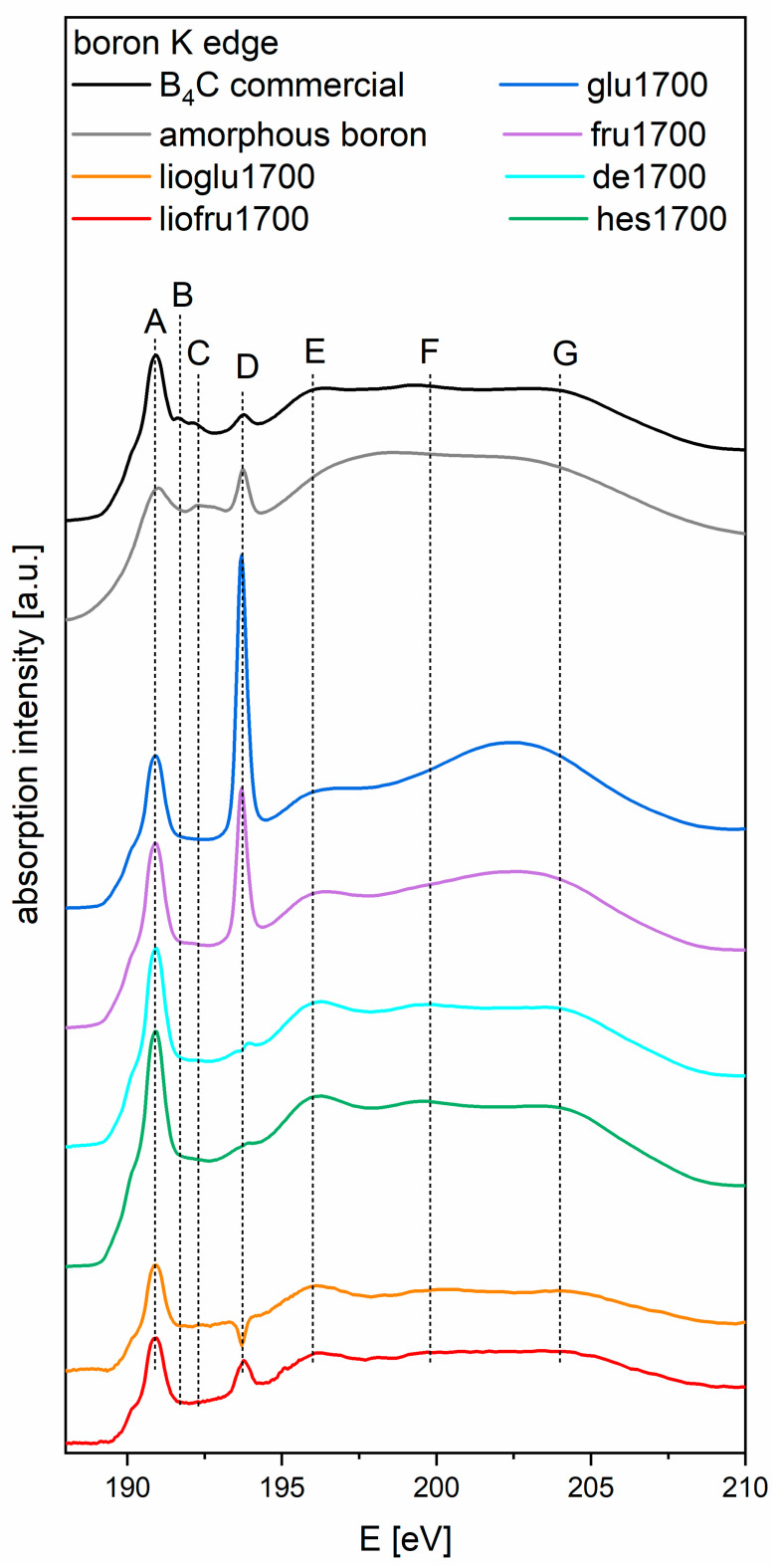
Boron K edge XAS spectra for powders of boron carbide obtained from recrystallized and freeze-dried precursor after pyrolyzing at 850 °C and heat treatment at 1700 °C (spectra in color) and reference spectra of commercial B_4_C (black) and amorphous boron (gray). Vertical lines indicate the characteristic spectral features for the B_4_C reference sample.

Figure 6 presents the K edge boron XAS spectra for powders of boron carbide obtained from recrystallized and freeze-dried precursor after pyrolyzing at 850 °C and heat treatment at 1700 °C, as well as two reference spectra: commercial B_4_C and amorphous boron. By comparing with Figure 4 in reference [22], we can see the spectrum of pure B_2_O_3_, with its characteristic peak at 193.7 eV, for all samples. Its position is slightly different than that stated in the cited article (194 eV) due to the difference in energy calibration between the experiments (based on reference [20]) and the data shown in reference [22]. Spectra of all samples have a similar shape (glu1700 and fru1700 differ above 197 eV), displaying the characteristic spectral features of the B_4_C reference sample, especially peak A at 191.2 eV (π* states). Features B (191.98 eV) and C (192.6 eV) were not present in the samples. Feature D (194 eV) indicates the presence of B_2_O_3_; as the spectra are normalized, the height of the feature indicates the amount of boron oxide present. The shape of the spectra above 195 eV is slightly different for both samples where the precursor saccharide was HES. The use of saccharides for the carbothermic reduction of boron carbide probably increased the melting point of boric acid and influenced the formation of new bonds between the saccharide and boric acid, which affected the synthesis of boron carbide. The presence of a depression instead of a peak at 194 eV in sample lioglu1700 was caused by subtraction of the spectra measured on carbon tape from the very weak signal provided by this sample. Features E (196.3 eV), F (199.8 eV), and G (204.3 eV) (σ*-like states) were present in all the samples, except for glu1700 and fru1700, which lacked features F and G. In summary, all obtained materials, regardless of their preparation method, contained the B_4_C phase, which was probably surrounded by a shell of boron oxide.

**Figure 7:**
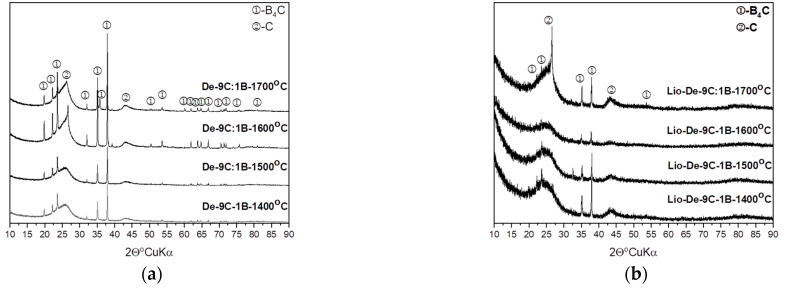
The X-ray patterns of the powders prepared from the mixtures of dextrin: (**a**) recrystallized and (**b**) lyophilized.

An XRD analysis confirmed the presence of three phases in the obtained powders from samples heat-treated at a temperature of 1300 or 1700 °C: boric acid (ICSD 98-002-4711), graphite-like structure (ICSD 98-002-4711), and boron carbide with stoichiometry close to B_13_C_2_ (ICSD 98-006-8152). The size of boron carbide was calculated using the Scherrer formula along the (021) direction. Figure 7 shows the XRD patterns of dextrin mixed with boric acid with a molar ratio of carbon to boron of 9:1, prepared using both methods and synthesized at 1400 to 1700 °C. Upon comparing the recrystallized and lyophilized diffractograms of the same weight ratios synthesized at the same temperature, we observed a significant difference between the XRD patterns. Powders that were recrystallized had much narrower and less blurry reflections compared with lyophilized samples, in both freeze-dried and recrystallized samples, and we observed a raised background, which indicated the presence of amorphous material; a little more amorphous material was present in the freeze-dried samples than in the recrystallized samples.

**Figure 8:**
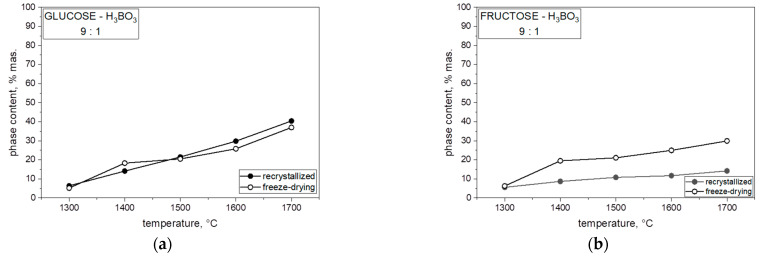

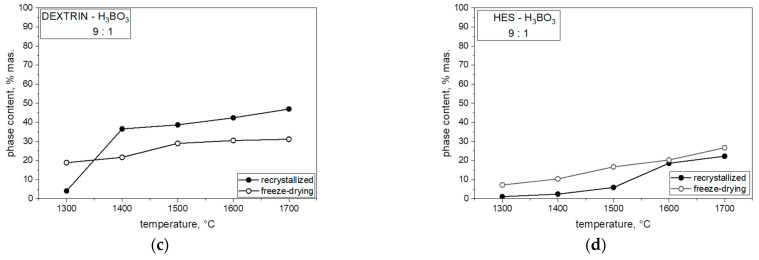
Temperature dependences of boron carbide content in the prepared powders from recrystallization and freeze-drying: (**a**) glucose, (**b**) fructose, (**c**) dextrin, and (**d**) HES.

Figure 8 presents the percentage of boron carbide (B_13_C_2_) phase in the heat-treated powders for each of the mixtures of saccharides and boric acid. In each case, both with the varying molar ratios of carbon to boron and the different saccharides, which were freeze-dried or recrystallized together with boric acid, an increase in the B_13_C_2_ phase content in the obtained powder can be observed with an increase in synthesis temperature. The highest content of boron carbide in the obtained powders was observed at 1700 °C, related to the expansion of boron carbide grains. The content of boron carbide in the obtained powder significantly influenced the comparison of the saccharide precursor used. When we analyzed the results for two monosaccharides, glucose and fructose, in the same proportions, a higher content of B_13_C_2_ phase at 1700 °C in lyophilized and non-lyophilized samples was synthesized with glucose. The difference in B_13_C_2_ phase with an increase in temperature and boron ratio was visible in all samples with different percent ratios.

**Figure 9:**
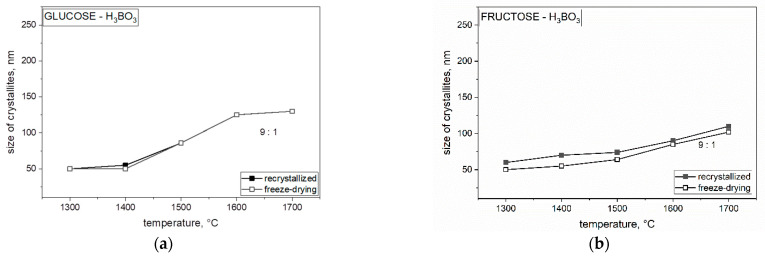

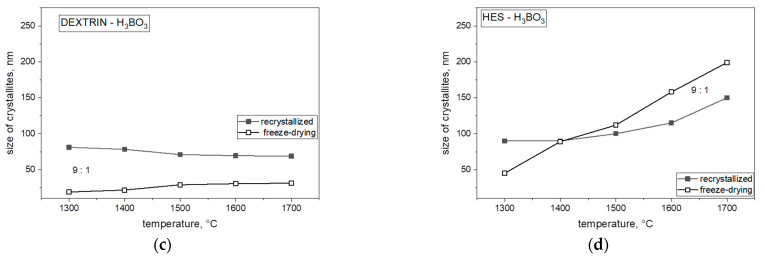
The changes in the crystallize size of boron carbide obtained with different precursors mixed with boric acid with a molar ratio of 9 C:1 B using both methods and heat treatment from 1300 to 1700 °C: (**a**) glucose, (**b**) fructose, (**c**) dextrin, and (**d**) HES.

Analyzing the obtained results in Figure 9, we concluded that the size of crystallites with the use of both freeze-dried and non-freeze-dried saccharides is significantly influenced by temperature. The crystallite size for each weight ratio and both freeze-dried and recrystallized precursor increased significantly at 1700 °C and varied with each saccharide. By comparing each saccharide with each other, we found that lyophilized polysaccharides had the smallest size of crystallites compared with the same recrystallized polysaccharides. Further particle size increase in boron carbide (B_4_C) may be related to the presence of boron oxide and the mechanism of transfer of individual components through the liquid phase. The Ellingham diagram shows the Gibbs free energy of oxide formation, indicating that the direct reaction between carbon monoxide and boron oxide should occur at a much higher temperature, around 1600 °C. The temperature of boron carbide was lower than as shown in Figure 8 and Figure 9, especially at 1300 °C, suggesting that at least some of the boron was linked to carbon by chemical bonds with carbon in the precursor after pyrolysis, which could become the nucleus of boron carbide crystallization (B_4_C) below 1600 °C. The formation of a nucleus of crystallization at a lower temperature was confirmed by spectroscopic studies (MIR). The further particle size (Figure 8) increase in boron carbide (B_4_C) may be related to the presence of boron oxide and the mechanism of the transfer of individual components through the liquid phase.

**Figure 10:**
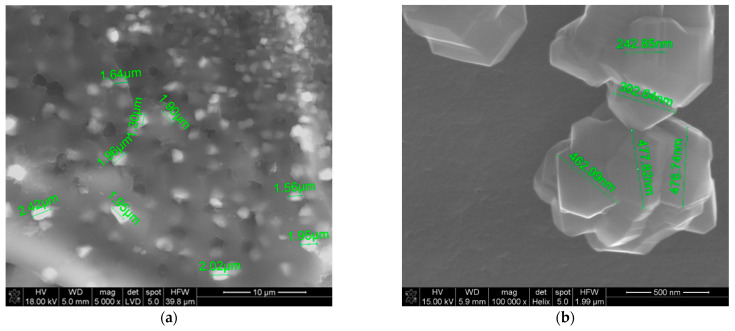

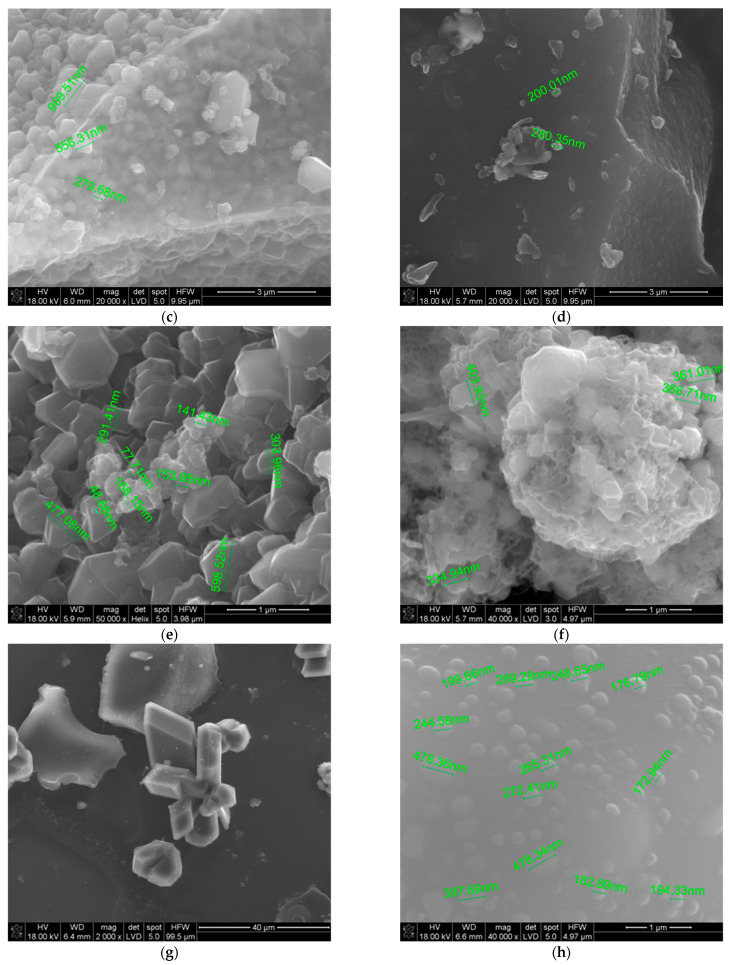
SEM images of the boron carbide obtained from powders prepared at the same temperature using saccharides: (**a**) glucose, (**b**) freeze-drying glucose, (**c**) fructose, (**d**) freeze-drying fructose, (**e**) dextrin, (**f**) freeze-drying dextrin, (**g**) HES, and (**h**) freeze-drying HES [16].

By analyzing the SEM images in Figure 10 of the powders obtained from polysaccharides, we concluded that the selection of the saccharide determines the size and morphology of the powders obtained as a result of the synthesis of polysaccharides. Comparing the two monosaccharides with each other, we concluded that the powders obtained from recrystallized glucose are much smaller than the recrystallized fructose as a precursor (Figure 10a–d). We found that the particle size, when used as a glucose saccharide precursor, ranged from 200 nm to about 1 μm, whereas for fructose, it ranged from 2 to 4 μm. The differences between the two monosaccharides are probably due to the presence of an aldehyde group (-CHO) in glucose, which is an aldose, whereas fructose is a ketose and we have a ketone group (-CO). In the case of polysaccharides, the best results were obtained for powders produced from dextrin, where the particle size for the recrystallized powder ranged from 150 to 600 nm, whereas for the recrystallized HES precursor, it ranged from 10 to 20 μm (Figure 10e–h). Comparing the particle sizes obtained from the same weight proportions of carbon to boron, we noted that, despite the same reaction and the same temperature being used, the selection of the precursor significantly affected the size of the particles obtained.

The findings from this study imply that freeze-drying of a saccharide precursor mixed with boric acid influences the morphology of the obtained boron carbide, the B_13_C_2_ phase content in the obtained powder, and the size and grain of the crystallites. The presented results indicate that the selection of precursors and the conditions of their heat treatment can be used to control the morphology of boron carbide powders. The current problem is the removal of excess carbon from the system and the fragmentation of boron carbide aggregates, which is the subject of current research. We note that our research has two limitations. The first one is the type of saccharide precursors used, which determines the concentration of boron carbide in the obtained powders. The second limitation is the too-pure powders from the graphite-like structure. Despite attempts to remove excess carbon from the obtained powders after saccharide synthesis from the 9 C:1 B molar ratio in the case of thermal oxidation, boron carbide oxidizes first, on the surface of which boron oxide is formed as a result of the oxidation of B_4_C particles, while the graphite-like structure does not affect the powders obtained. The only solution is intensive grinding and crushing of the obtained powders.

We confirmed that the most important factor influencing the particle size is the synthesis temperature, and the highest maximum synthesis temperature for boron carbide nanoparticles is 1600 °C because, above this temperature, we observed a significant increase in the size of the crystallites.

## 4. Conclusions

Our work led us to conclude that the morphology and size of boron carbide strongly depend on the saccharide precursor and the temperature of synthesis. Summarizing the obtained results, it can be stated that the reaction temperature has the greatest influence on the particle size of the obtained boron carbide (B_13_C_2_). As the temperature increases, crystallite growth takes place and the particle size increases, thus increasing the agglomeration and aggregation of boron carbide (B_4_C) particles. Lyophilization of saccharide precursors reduces the size of the particles and allows to obtain fine boron carbide, but the lyophilization process itself causes, for most compositions, a decrease in the percentage of B_13_C_2_ phase in the obtained powders, compared to recrystallized powders. The use of saccharide precursors for the carbothermic reduction of boron carbide increases the melting point of boric acid and influences the formation of new bonds between the saccharide and boric acid, which affect the synthesis of boron carbide. The type of saccharides precursors used determines the size of the crystallites, morphology, and the agglomeration and aggregation of boron carbide (B_4_C). The best results are obtained from dextrin (100–400 nm, both for lyophilized and recrystallized samples). Compared to the second used polysaccharide, we can see a significant influence of the saccharide precursor on the B_4_C particle size, because using hydroxyethyl starch (HES) as a saccharide precursor, we obtain particles larger than 10 to 20 μm.

## Figures and Tables

**Table 1:** Position of the D- and G-bands, their intensity, and I_D_/I_G_ ratio for pure saccharides and the precursors recrystallized, freeze-dried, and pyrolyzed at 800 °C for 1 h [16].

Sample	D Band (cm^−1^)	G Band (cm^−1^)	I_D_/I_G_
Glucose	1376	1596	0.44 [16]
Glucose—H_3_BO_3_, recrystallized	1358	1599	1.12 [16]
Glucose—H_3_BO_3_, freeze-dried	1357	1602	1.01
Fructose	1351	1597	0.79 [16]
Fructose—H_3_BO_3_, recrystallized	1353	1592	1.28 [16]
Fructose—H_3_BO_3_, freeze-dried	1358	1600	1.29
Dextrin	1362	1593	0.68 [16]
Dextrin—H_3_BO_3_, recrystallized	1352	1590	1.01 [16]
Dextrin—H_3_BO_3_, freeze-dried	1356	1599	1.15
HES	1356	1593	0.87 [16]
HES—H_3_BO_3_, recrystallized	1359	1593	1.21 [16]
HES—H_3_BO_3_, freeze-dried	1358	1597	1.30

**Table 2:** The parameters of lines on the ^11^B MAS NMR spectra of different saccharide precursors mixed with boric acid.

Sample	^11^B Position (ppm)	^11^B Relative Intensity (%)
Glucose, recrystallized	−19.4	100
Glucose, freeze-dried	−18.8	100
Fructose, recrystallized	−18.7	100
Fructose, freeze-dried	−16.4	100
Dextrin, recrystallized	−16.7	100
Dextrin, freeze-dried	−19.5	100
HES, recrystallized	−20.4	100
HES, freeze-dried	−19.9	100

## Data Availability

The data presented in this study are available on request from the corresponding author.
